# “We Just Did Not Get on”. Young Adults’ Experiences of Unsuccessful Psychodynamic Psychotherapy – A Lack of Meta-Communication and Mentalization?

**DOI:** 10.3389/fpsyg.2020.01243

**Published:** 2020-06-17

**Authors:** Camilla von Below

**Affiliations:** Clinical Division, Department of Psychology, Stockholm University, Stockholm, Sweden

**Keywords:** unsuccessful treatment, psychodynamic psychotherapy, young adults, grounded theory, secondary qualitative analysis, mentalization, meta-communication

## Abstract

In order to avoid suboptimal psychotherapy, research needs to highlight and analyze obstacles in such treatments. This clinically oriented article brings together empirical material of unsuccessful psychotherapy with young adults; empirical material on the therapists’ views of the same therapies; and theoretical perspectives on mentalization, therapeutic alliance, and young adulthood. Through a secondary qualitative analysis, it presents a tentative process model of how suboptimal psychotherapy with young adults develops, how it could be handled clinically, and possibly prevented. In three studies, experiences of young adult patients (aged 18–25; *n* = 27), in psychoanalytic therapy at an outpatient clinic, who did not improve from therapy (defined as no reliable and clinically significant symptom reduction) and/or were dissatisfied, and their therapists, were analyzed. Patients described experiences of not being understood and not understanding therapy, whereas therapists described patient non-commitment. These results were compared from the developmental perspective of mentalization in young adulthood. The primary grounded theory analyses and secondary analysis resulted in a tentative process model of the development of suboptimal psychotherapy with young adults. Suboptimal therapy is described as a vicious circle of therapist underestimation of patient problems, therapeutic interventions on an inadequate level, and diverging agendas between therapist and patient in terms of therapeutic alliance, resulting in pseudo-mentalizing and no development towards agency. A benign circle of successful therapy is characterized by correct estimation of patient problems, meta-communication, and the repair of alliance ruptures. One clinical implication is that therapists of young adult patients need to establish verbal and nonverbal meta-communication on therapy progress and therapeutic alliance. The importance of the patients’ present mentalization capacity and adjusted interventions are demonstrated in an example. Research in the field should be process-oriented and investigate the effect of meta-communication and interventions targeted to foster therapeutic alliance based on this theoretical model, particularly for young adults.

## Introduction

Psychodynamic psychotherapy is helpful for adults, adolescents and children with various psychological problems (Barber et al., 2013; Lambert, 2013b) but does not help every individual. The awareness that certain subgroups and individuals might need different approaches in therapy is now leaving its mark on psychotherapy research, with in-depth studies on what works for whom (Zilcha-Mano, 2019). One conclusion is that a number of patients stay in treatment although it does not seem to be helping them (Lambert, 2013a), thus spending their own and their therapist’s time and effort for very little benefit. It has been pointed out that non-responding possibly deprives the patient of the opportunity to have a successful treatment elsewhere (Dimidjian and Hollon, 2010). It could also be added that it deprives the therapist of the chance to offer other patients more successful therapy, since the same therapist often has both successful and less successful cases (Wampold and Brown, 2005).

As a researcher and a clinical psychologist and psychotherapist, I am aware of the time and effort many clinicians spend in supervision trying to understand patients for whom treatment does not seem useful. It would be of great value to researchers and clinicians alike to know what makes patients stay in treatments that do not help them and how such therapies could be prevented, either by turning the deadlock in therapy into a productive process, or by singling out therapies which might not be helpful from an early stage.

To investigate unsuccessful psychotherapy in which patients stay for a substantial time (as opposed to dropping out), the first question is how non-improvement should be defined. The reliable change index (RCI; Jacobson and Truax, 1991) is often used for identifying cases without improvement, based on symptom levels before and after treatment. However, it needs to be taken into account that a high symptom level at the termination of therapy might not be due to therapy alone but other circumstances (Bowie et al., 2016), and that qualitative and quantitative measures do not always coincide in deciding whether psychotherapy was successful (von Below, 2017). Patients and therapists might have highly diverging views of the very same psychotherapy process and results (Dimidjian and Hollon, 2010; Gold and Stricker, 2011; Kächele and Schachter, 2014). Thus, the area is best investigated from different viewpoints and well defined in each study. To combine different measures is also an advantage, or have studies look at the same cases but with different methodological starting points (i.e., qualitative, quantitative, mixed method, meta-analyses, and secondary qualitative analysis) as it gives a fuller picture, e.g., when a patient shows deterioration in one measure and improvement in another.

However non-improvement is defined, we could expect a heterogeneous collection of factors contributing to it, including patient factors (Barber et al., 2013), circumstances outside of therapy (Bowie et al., 2016), and therapeutic alliance (Zilcha-Mano, 2017). Considering the diversity of factors that could be a part of unsuccessful psychotherapy, an explorative and inclusive approach is often needed, in which qualitative methods are useful (Barlow, 2010 Malterud 2001a).

Patients stress the importance of a good emotional bond as part of treatment (Midgley et al., 2014b; Levitt et al., 2016). This has spurred research into particular challenges to form an emotional bond to certain groups of patients, among them young adults (aged 18–25). Clinicians have expressed that they use a particular approach with young patients, in which the therapeutic alliance and in particular the emotional bond is central, but needs more focus than when treating older adults (e.g., Paulson and Everall, 2003 Bury et al., 2007; Cooper, 2009; Binder et al., 2011; Lynass et al., 2012; Henden Sagen et al., 2013; Midgley et al., 2014b; for an overview, see von Below, 2017). From a developmental perspective, young adulthood is for most individuals a period of insecure employment, sudden changes, identity exploration, and many possibilities (Arnett, 2014). Clinicians need to take into consideration the instable life situation as well as the life decisions in young adulthood, in order to form a good therapeutic alliance with young adults (von Below, 2017). Since a strong therapeutic alliance is associated with good outcome (Horvath et al., 2011), using therapeutic interventions for forming good alliance with young adults is of importance. Young people are an age group in which mental health is deteriorating in Sweden, particularly expressed in psychosomatic symptoms, anxiety and depression (Public Health Agency of Sweden, 2018), which further enhances the importance of treatments adjusted to the age group.

Patients’ own explanations of why treatment was unsuccessful give researchers and clinical therapists hypotheses on how therapy can be better presented to patients, and how therapy progression can be followed, but is under-used as a source of information on how to improve therapeutic technique (Bohart and Wade, 2013; McLeod, 2013; Midgley et al., 2014a). In line with this, four empirical studies leading to this present study have focused on negative experiences among young adults in psychoanalytic psychotherapy and their therapists’ view of the same therapies (von Below et al., 2010; von Below and Werbart, 2012; Werbart et al., 2015, 2018). One result was that patients experienced limitations in the therapeutic relationship, which made them restrained in therapy. Another was that therapists attributed the limited outcome in therapy to patients, whereas patients experienced a lack of therapist commitment as well as misdirected therapeutic actions important aspects of bad outcome. Their different perspectives could be understood in the light of limited therapeutic alliance (Bordin, 1979; Zilcha-Mano, 2019) by not sharing goals of therapy and an emotional bond that had severe limitations from the patients’ view. The results were interesting in themselves, and other studies reporting patients’ expectations and experiences of psychodynamic psychotherapy (Rennie, 2002; Midgley et al., 2014a), including meta-studies (Levitt et al., 2016) have come to similar conclusions on the importance of a positive therapeutic relationship and shared the goals of therapy. However, the clinical usefulness of the results can be further enhanced if the results are analyzed beyond the level of what patients say, by comparing patient statements to their therapists’ reports of the same therapies, as well as placing the therapies in the context of the patients’ present life situation and capacity and exploring the implications of this for the therapeutic stance, which is the focus of this present study.

The aim of the present article is to draw theoretical and clinical conclusions on the process of suboptimal psychodynamic psychotherapy from the young adult patients’ and therapists’ view, leading to clinical advice, in order to avoid suboptimal outcome. In line with the grounded theory approach, the study is explorative and empirical in its starting point. It analyses (1) what leads to suboptimal outcome according to the patients and therapists; (2) what the combined picture of these two perspectives tell about the therapeutic process; and (3) how the therapeutic process can be understood theoretically in the light of the concept of young adulthood and mentalization. “Suboptimal” is defined in this article as psychotherapy which either does not reach the goals decided by patient and therapist or leaves the patient dissatisfied (see each study for details) regardless of whether goals were reached according to the RCI (Jacobson and Truax, 1991).

The conclusions are drawn from cases which were considered unsuccessful either by self-report measures (Werbart et al., 2015) or qualitative measures (von Below and Werbart, 2012) and their therapists’ view of the same therapies (Werbart et al., 2018), which will be compared to cases with an average outcome (von Below et al., 2010) in a secondary qualitative analysis (Heaton, 2008). The tentative process model of the way to suboptimal outcome, as well as ways to break the negative spiral into suboptimal outcome has been published in my doctoral thesis (von Below, 2017), but is presented here along with a more extensive theoretical analysis and clinical conclusions. The secondary analysis adds a theoretical interpretation to the data, although the analysis in itself is not deductive or theory-driven.

## Materials and Methods

The present study is thus a secondary analysis (Heaton, 2008) of qualitative data from four studies with a focus on the hindering factors in psychodynamic psychotherapy. It should be seen as an additional qualitative interpretation. In two of the studies, the experiences of young adult patients in suboptimal psychodynamic psychotherapy were investigated (von Below and Werbart, 2012; Werbart et al., 2015), and in the third, focus was on the experiences of therapists of patients in suboptimal psychotherapy (Werbart et al., 2018). A fourth study investigated the experiences of young adult patients with depression diagnosis and average therapy outcome in the same larger study (von Below et al., 2010).

A secondary analysis of qualitative data can be used to aggregate or re-use data in order to answer questions not addressed in the primary studies (Heaton, 2008). The present secondary analysis re-uses data (interviews) as well as codes and categories from the grounded theory analyses in the primary analysis. By combining data from patient and therapist interviews in the primary analyses, discrepancies in their view of the therapeutic process were observed within the framework of each study. The conclusions and discussions in each study were limited to the focus of that particular study. When considering the conclusions from all four studies taken together, I observed that a re-analysis might contribute with new patterns and themes. The secondary analysis is thus a re-analysis and a synthesis of the primary studies with a particular focus on the theoretical understanding of the results (Heaton, 2008). The secondary analysis included further theoretical perspectives in line with the grounded theory approach in which the exploration of the empirical findings starts with a minimum of references to other research and theories but is added in the discussion section to present the process model grounded in data (Charmaz, 2014). The secondary analysis is thus still inductive but adds and stresses a theoretical framework as an interpretation.

### Setting

The studies were conducted within the Young Adults Psychotherapy Project (YAPP), a longitudinal, naturalistic study of young adults (aged 18–25) in psychotherapy at the former Institute of Psychotherapy in Stockholm, Sweden. The patients in the project as a whole reported low self-esteem (97%), conflicts in close relationships (66%), depressed mood (66%), and anxiety (55%) (Wiman and Werbart, 2002). Moreover, about one-third of the patients had personality disorders according to the *DSM-IV and ICD-10 Personality Questionnaire* (DIP-Q; Ottosson et al., 1998). The therapies (mean duration 22.3 months, SD = 17.2) were aimed at improving the patients’ ability to manage developmental strains and not manualized. Duration, frequency (once or twice weekly), and goals were jointly formulated by patient and therapist at the beginning of therapy. Treatment outcomes were studied at termination, after 1.5 years, and at a 3-year follow-up (Philips et al., 2006; Lindgren et al., 2010). Generally, there were large improvements on a group level in global functioning (Lindgren, et al., 2010).

The psychoanalytically trained therapists (*n* = 37) had backgrounds as psychologists, psychiatrists, and social workers before they started their employment as psychotherapists, supervisors, and teachers at the institute. They met weekly in clinical teams, where treatment problems were discussed, and had access to supervision. Adherence could not be measured.

### Participants

From the Young Adult Psychotherapy Project, a subsample of participants was used in each primary study in accordance with the aim of that study. The subsamples are described below.

The secondary analysis comprised all of the participants from the primary studies. Due to an overlap of four patients, who took part in more than one of the primary studies, and one of them in all three, the total number of patients were 39 and therapists were 8.

#### Dissatisfied Psychotherapy Patients (*n* = 7)

The first study (von Below and Werbart, 2012) included all patients in the project thus far who were dissatisfied with individual psychotherapy, defined by the qualitative criterion that they expressed dissatisfaction with therapy in the termination interview. von Below read the 70 interviews available from termination and 59 from follow-up, listing those predominantly dissatisfied with therapy, defined as expressing more dissatisfaction than satisfaction with therapy. Seven clear cases and three possible cases were found; all ten were discussed in the research team (Werbart and four other researchers) and seven patients (six women, one man) were labeled dissatisfied. Four of these had personality disorder diagnoses according to DSM-IV (American Psychiatric Association, 2000) and four had axis 1 diagnoses: acute stress syndrome, adjustment disorder with depressed mood, mood disorder due to medical condition, and major depressive disorder (recurrent). Therapy length was varying (2–48 months, *M* = 16.9).

#### Non-improved Psychotherapy Patients (*n* = 20)

The second study (Werbart et al., 2015) included all patients who did not improve significantly from individual therapy, i.e., who both belonged to the clinical range pre-treatment and showed deterioration or no symptom reduction at termination of psychotherapy. Of the 20 patients, 17 (85%) were women. The pre-treatment symptom level was measured by the Global Severity Index (GSI) of the Symptom Checklist-90-R (Derogatis, 1994). Change was measured using the RCI (Jacobson and Truax, 1991). For research design reasons, only nine had been diagnosed in accordance with the DSM-IV (American Psychiatric Association, 2000). Two patients had dysthymia and personality disorder not otherwise specified (NOS), one mood disorder due to medical condition, one obsessive-compulsive disorder, one acute stress disorder, one anxiety disorder NOS, and two personality disorder NOS.

#### Therapists of Non-Improved Patients (*n* = 8)

Study three included the therapists (Werbart et al., 2018) of the non-improved patients in study two. Due to research design, not every patient’s therapist had been interviewed. The seven therapists included treated eight patients. Four therapists were female, three male; two were social workers, four psychologists and one psychiatrist. Six therapists were senior licensed psychotherapists with 6–14 years of experience and one had basic training in psychodynamic psychotherapy.

#### Patients With Depression (*n* = 17)

Study four (von Below et al., 2010) included all patients who were diagnosed with a depression diagnosis according to DSM-IV (American Psychiatric Association, 2000), at the beginning of therapy within the YAPP project. Nine of the patients were enrolled in individual psychotherapy with a mean duration of 27 months (range 14–48), eight in group therapy in three different groups, with mean duration 15.5 months (range 7–27 months).

### Material

Interviews were conducted at therapy termination and at follow-ups at 18 and 36 months after termination. The interview protocol comprised the private theories interview (PTI; Werbart and Levander, 2005) and Object Relations Inventory (ORI; Blatt et al., 1979; Gruen and Blatt, 1990). The PTI is semi-structured and collects narratives on problem formulations, ideas of background, ideas of cure, descriptions of changes, and retrospective views of what could have been different. The ORI focuses on the participants’ descriptions and views of significant others and themselves by asking participants to describe their closest relations and their therapists, followed by exploration of the answers. The interviews lasted 60 min and were recorded and transcribed verbatim. The interviewers were psychotherapists and researchers at the Institute of Psychotherapy trained in the PTI and ORI interview techniques.

### Analysis

#### Primary Analysis

The qualitative method GT (Fassinger, 2005; Rennie, 2006; Charmaz, 2014) designed for analyzing interview material without preconceived categories was used in the primary studies. GT aims at generating tentative conceptual models grounded in empirical data and is often considered the method of choice when studying interactive, reciprocal processes and underexplored fields of knowledge. GT is especially useful for analyzing processes, or interrelations. We followed the steps outlined by Strauss and Corbin (1998) and developed by Charmaz (2014):

1. *Open coding*: the transcribed interviews were read line by line and all units of meaning were labeled with words or sentences capturing the participants own words. To reduce the risk of letting the researchers’ preconceptions interfere with the initial codes, there was a *constant comparative analysis* against data and across coders. Codes were merged, defined and grouped together in preliminary categories, which were further defined.
2. *Axial coding*: the analysis moves from the descriptive to the theoretical. Focus shifts from individual codes to patterns (temporal, causal, and theoretical) in the relations between categories, leading to a number of categories and one or a few main categories, all connected by well-defined relations. Possibly, a core category that theoretically summarizes the material is formulated.
3. *Selective/theoretical coding*: the process model that was created in the axial coding created a need for further analysis of the empirical material or other patterns in data, which prompted a return to data.

Computer programs were an aid in the overview of codes, quotations, categories and patterns. We also used *memo-writing* to conceptualized material on an early stage (Charmaz, 2014).

#### Secondary Analysis

Codes and categories from the primary studies were revisited by the author of the present article. In some cases, interviews or excerpts from the interviews were re-read in order to define categories and codes with the new research question. No new data were collected. The process is best described as an *amplified supra analysis* of pre-existing data (Heaton, 2008), in which two or more existing datasets are combined or compared in order to explore a partly new research question that transcends the aim of the primary analysis. It could not be described as a fully conducted qualitative meta-analysis, as the studies are part of the same project. However, the method in the secondary analysis is similar to that of a qualitative meta-analysis as it creates new themes and a further theoretical understanding by aggregating qualitative data (Heaton, 2008). In line with grounded theory, the aim of the amplified supra analysis was to present a tentative process model of suboptimal psychodynamic psychotherapy with hypotheses on how this could be prevented. The interpretation is grounded in data and to a certain degree hermeneutic as it is an interpretation of the process of suboptimal therapy with the intention of understanding it (Rennie, 2006). The aim is not to confirm causality but rather to explore the area to propose hypotheses for further research.

The analysis was carried out by the author and discussed with the co-writer of the primary studies, Andrzej Werbart, and research teams at Stockholm University.

#### Researcher Reflexivity

The researcher’s preconceptions, background, theoretical preferences, and experiences inevitably affect the interpretation of data. No attempt to put one’s knowledge and preconception into brackets will be complete. Thus, reflexivity is necessary for transparency. I joined the larger project of which this study is one part in 2006 as a newly graduated clinical psychodynamic psychologist with theoretical knowledge of psychotherapy, but little experience. With increasing clinical experience, doctoral studies, and as a lecturer of psychodynamic psychotherapy I have continued to combine clinical and theoretical knowledge on mentalization, attachment, and affect focused psychotherapy which has influenced my analysis in the present study, possibly by drawing my attention to aspects in data central to mentalization. It is both a limitation and a strength. The limitation is that other perspectives might play a lesser part. On the other hand, the project leader and co-researcher professor Andrzej Werbart has a psychoanalytic training which brought other perspectives into the discussion of the analyses. To have knowledge of mentalization theory and practice is also a strength, as it makes research clinically useful.

My own experiences of conducting successful and less successful therapies have possibly deepened my understanding for the research material. I have also experienced the importance of the therapeutic alliance in everyday work. However, I might also have lost some of the naivety that comes with being less acquainted with a field of research. In the beginning, I could not fill the gaps in the analyses with my own preconceptions of therapy. This might be the case in the later studies, no matter how hard I have tried to avoid this and to achieve triangulation and discussions with other researchers.

## Results

Here follows a summary of the primary results of each study, with a concluding comparison of the studies. The italicized words refer to categories in the process models presented in the original articles.

### Dissatisfied Psychotherapy Patients

The dissatisfied patients described a vicious circle of dissatisfaction, summed up in the core category as *abandonment with their problems*: an experience of being abandoned with their problems in ways elaborated by the subcategories. Participants described *not being understood* when therapists were inattentive, uninterested, or not focusing on what participants considered important – *the therapist went her own way*. Participants generally described an *unsure, critical, powerless therapist* and experienced *lack of therapist response* and *lack of confidence*. One variant was *therapist absent or had problems of her own*, implying non-stability.

The core categories *insufficient flexibility and intensity* and *absent links to everyday life* summed up and interpreted how participants described *wanting advice, answers, and practical exercise* and *wanting direction* in therapy. Most patients expressed a wish for a therapist who structured the sessions better. The variant *feeling unable to reach or express own feelings* was the only category focusing on patients’ own inability. Patients generally saw the therapy method and the therapist as the main obstacles to successful therapy.

Based on negative experiences, the participants generally concluded that *therapy ended too early*, *therapy did not help*, *needing some other kind of help* and as a variant, *therapy made things worse*. One patient expressed at termination: “Now I feel all shut up inside myself. It feels worse” (von Below and Werbart, 2012).

All participants mentioned some positive aspects. Typically, *therapy provided some acceptance and insight into oneself and one’s problems* and *it felt good to talk*. *The therapist was gentle, sensitive, and stable* but this was vague and could not be exemplified.

### Non-improved Psychotherapy Patients

The core category *spinning one’s wheels* summed up the experience of continuing without getting anywhere. Six categories pointing toward the core category explained positive and negative experiences of therapy balancing each other. Positive experiences of some symptom reduction and being in therapy with a listening, professional and wise therapist, who sometimes confronted the patient and reflected about what was said in a helpful way, outweighed negative experiences of a distanced relationship, too much focus on understanding and unchanged core problems. One patient said in retrospect: “When I think back on the therapy, I get the feeling that I often sat and talked; sometimes something important came up, but often it felt like it was pretty much just spinning my wheels” (Werbart et al., 2015).

As time passed, outcomes of therapy became clear in four subcategories. Generally, instead of helpful therapy, participants described their *own helpful activity*, e.g., moving to a new place as bringing positive change, as well as *mending life conditions*, such as support from relatives or friends. As a variant, *negative impacts of life events* were neither caused by therapy, nor did therapeutic experience help resolve them.

Generally, *therapy generated some improvements* but *therapy was insufficient* and there were *remaining core problems*. Typically, participants described *impaired emotional life* for which therapy was not to blame, but also not helpful.

### Therapists of Non-improved Patients

The conclusive experience of the therapists was summed up in the core category *having half of the patient in therapy*. Initially, the therapists experienced a *stimulating collaboration*, at the same time as a *distance in the therapeutic relationship*. However, the negative process developed and dominated at termination. The therapist experienced that the patient reacted with aversion to emotional, therapeutic closeness and the therapist experienced struggle and loss of control in therapy. The therapists described therapy outcome as favorable in the form of increased insight and mitigated problems, while core problems remained. This split picture was interpreted as a sign of a pseudo-process emerging when the therapist allied herself with the patient’s capable and seemingly well-functioning parts. The therapists’ experiences could be compared to the non-improved patients’ “spinning one’s wheels” in therapy. The therapists seemed not to have succeeded in adjusting their technique to their patients’ core problems, despite attempts to meta-communicate.

One therapist summed up therapy at termination: “It reflects pretty much how her life is like. On the surface everything looks very competent and good. But you still have a sense that there is something going on under the surface. And I cannot get the hang of what’s going on there” (Werbart et al., 2018).

### Patients With a Former Depression Diagnosis

Participants with a depression diagnosis pre-treatment, who were in therapy with average outcome, described the process of finding themselves and a new identity as central, along with symptom relief. *Finding oneself* and *finding one’s way of life* were changes and contributors to change as the participants reported *feeling better*. They felt proud and confident in studies or work. Relationships brought joy and satisfaction. Participants described a new attitude to life with humor, courage and acceptance, *viewing life differently – doing differently*. Increasing self-knowledge was a prerequisite for this, but also a result of it. New experiences and changes in therapy contributed to positive change, such as the typical *sharing what’s inside oneself*. Talking and reflecting in a safe environment were experienced as helpful and as new abilities, as was the capacity to stand difficult feelings. *Gaining perspectives and understanding* through the therapist or therapy group members were helpful. *Therapy as a place and time for oneself* was important for many participants. *The march of time* and *other treatments* such as yoga were of help and so was anti-depressant medication.

Participants reported *feeling uncomfortable in therapy* from time to time, often attributing these shortcomings to themselves. *Wanting treatment to be different*, a need for advice, active guidance or longer therapy was common. Participants also brought up *problems in therapy* such as long holiday breaks. There were negative experiences that could impede the experienced changes or be an obstacle, but these could be alleviated by positive outcomes, for instance *getting stuck in problems* and *feeling worse* through medication, therapy, and life circumstances. *Finding it difficult to do things differently* despite intellectual knowledge of how to do so was reported as hindering. To conclude, obstacles and dissatisfaction with some aspects of therapy were common but could generally be overcome with time and effort.

### Summary of Results From the Primary Studies: A Comparison Between Dissatisfied Patients, Non-improved Patients, Their Therapists, and Patients With Average Outcome

Patients with an average outcome and an earlier depression diagnosis described their way to improvement as *finding oneself and one’s way of life*. They described a new understanding of their own needs and responsibilities, which facilitated their decisions in life and gave them a sense of having command in their own lives. They established themselves as self-aware agents who could act, rather than be left to the circumstances. This new ability and experience gave their life direction and symptom relief. They appreciated the warmth expressed by the therapist and therapy group, understanding, honest feedback, active interventions and advice as part of development.

Correspondingly, participants in suboptimal therapies called for therapeutic actions similar to these: active interventions, focus on questions in their lives that matter to the participant. They wished for a therapist who offered advice and explanations, was interested, and intensified therapy by confrontation if needed. Thus, participants in suboptimal therapy had an intuitive knowledge of what was lacking in therapy and might have been helpful. With one exception, they did not bring this up with their therapist.

At termination, participants in suboptimal therapies described that important problems remained, most of all due to the lack of therapist engagement and understanding. Participants wanted the therapists’ concern and guidance. Indeed, therapists in the study also felt interested and concerned about the patients. They wanted to, and tried to, help patients with their emotional suffering by offering a trusting therapeutic relationship, interventions, and confrontation, but perceived the patients as withdrawing or unwilling. Patients wanted confrontation and help to change from the therapists, still the therapists perceived the patients as unmotivated when offering exactly that, as expressed by therapists in the quote “*Having half of the patient in therapy*.” The question of how therapist and patient could aim for the same goal, but not find the means to do so, is intriguing and will be further discussed in the following.

When reviewing the interviews of patients in suboptimal therapy, it became clear that the patients in suboptimal therapies expressed themselves in a way that differed from the patients in therapies with average outcome. Their wishes for advice, explanations of one’s own behavior, and the attempts to understand the therapist were generally remarkably concrete and lacked the reflection interviews in a study of patients with average outcome showed. It was also reflected in their relatively high percentage of personality disorders. The concrete understanding of others’ intentions and thoughts could indicate that the participants were mostly in pre-mentalizing modes (that is teleological thinking, psychic equivalence, or pretend mode; Bateman and Fonagy, 2016) when trying to understand themselves and others, including their therapists.

## Secondary Analysis: Preventing Suboptimal Psychotherapy with Young Adults

**Figure 1:**
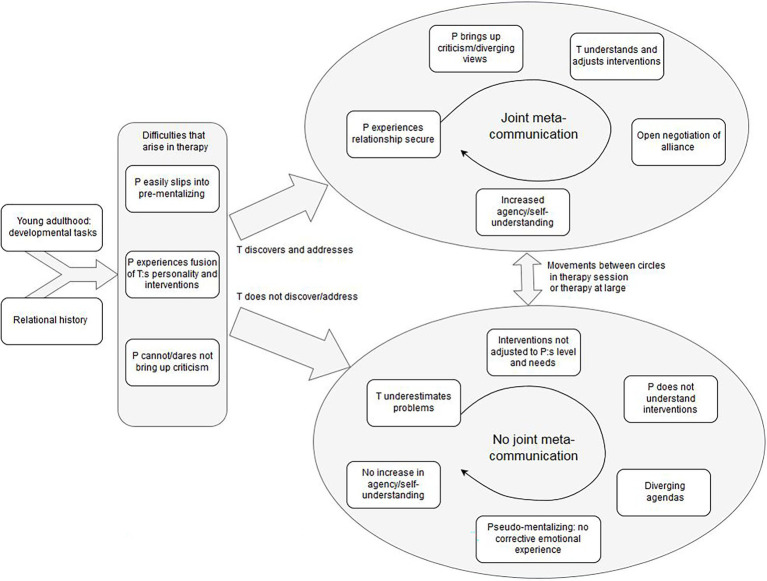
A tentative process model of suboptimal therapy with young adults, with emergent principles and hypotheses on how to prevent it.

The results and hypotheses from the second analysis are presented in the shape of a tentative process model (Figure 1). The model is an interpretation of the process of suboptimal psychoanalytic psychotherapy with young adult patients, understood from a developmental and relational perspective, based on the patients’ experiences as expressed in the interviews and theories of psychological development.

The model should be followed from left to right. The first box depicts *young adulthood: developmental tasks*, which gives the context for psychotherapy with young adults. Patients in the primary studies described how practical matters such as housing, employment and changing relationships were central in their lives and their psychological suffering. Beginning with Erikson (1959), developmental psychologists have pointed out the life stage specific challenges young adults in general face: to create a meaningful life with capacity for intimate relationships and independence from parents. Young adult patients are in a life stage that demands rapid decisions on how to form one’s life, decisions which will have future consequences. As pointed out by developmental psychologists (Arnett, 2014; Schwartz, 2016), such life decisions are both a part of becoming an adult and developing self-knowledge in order to create one’s own identity. Patients in the primary studies did not feel their therapists met their wish to reflect on their life decisions and own will in therapy. Instead, they their therapists stressed other aspects of the patient’s life, such as past relationship patterns to parents, which the patients did not see as important in the current situation. Thus, the means of therapy was not agreed on by the patient and therapist. The therapeutic alliance (Bordin, 1979; Zilcha-Mano, 2017) was impaired by this.

*Relational history* in the model refers to the patient’s attachment pattern and interpersonal functioning, which influence the therapeutic relationship. Patients with a low capacity for reflection in general, and limited experiences of secure relationships, naturally present a greater challenge for therapists when establishing a therapeutic alliance (Bateman and Fonagy, 2016) as well as in treatment, as it is a factor that is associated with low outcome (Barber et al., 2013). Treating patients with these difficulties demands knowledge and suitable interventions from the therapists. Among such relational difficulties is insecure attachment with negative and insecure internal working models (Bowlby, 1988) or low capacity for mentalization (Bateman and Fonagy, 2016). Zilcha-Mano (2017) refers to the reasonably stable way of relating to others, including the therapist, as the “trait-like” aspect of emotional bond in the therapeutic alliance. In the present study, the high percentage of personality disorders according to DSM-IV (American Psychiatric Association, 2000) among patients in suboptimal psychotherapy indicates that had relational difficulties and would have needed interventions targeted at these difficulties, i.e., targeted at the “trait-like” aspects of the ruptures in the therapeutic alliance (Zilcha-Mano, 2017), for instance negative internal working models in attachment terms (Bowlby, 1988). This will be discussed below.

The rectangle containing three boxes depicts the process in therapy. Young adult patients with relational difficulties and insecure attachment history face difficulties in pressing choices about housing and employment, as they have a less clear sense of their own wishes and directions in life. Their stress resilience is lower, which means they risk slipping into concrete modes of thinking (pre-mentalization, discussed below; Bateman and Fonagy, 2016) more often than a patient with relational security or less stressful life circumstances. The capture *patient easily slips into pre-mentalizing* depicts the concrete understanding patients express in the interviews of the present study, when interpreting their therapists’ actions in a concrete way, such as the change of hair color as a sign of the therapist’s psychological imbalance (von Below and Werbart, 2012). A permanent or temporary low mentalization capacity needs attention from the therapist and interventions intended to reduce anxiety and improve reflective functioning in the present (Bateman and Fonagy, 2016).

I suggest that theory of mentalization is helpful in understanding the negative therapeutic relationship described by patients in the study and to inform therapists of ways to improve the therapeutic alliance and avoid suboptimal psychotherapy. The definition of mentalization is the ability to understand that (and how) mental states including feelings, intentions, wishes, values, and goals in oneself and others underlie our own and others’ overt behavior (Allen et al., 2008; Bateman and Fonagy, 2016). The capacity develops gradually through childhood from a concrete understanding of others’ actions to a reflective stance, if the circumstances are supportive. The child reaches early a teleological stance, in which there is a rudimentary understanding of the rationality behind a certain behavior, but only with regard to concrete reality. In the psychic equivalence mode, the young child equates the internal state with the outside world, not experiencing its own feelings and states as representations of the external world, but rather the external world itself. The pretend mode, on the other hand, is the extreme separation of the internal and external world – mental states are not anchored in the external reality and thus not a representation, but imagination. In a fully mentalized mode, the individual is capable of keeping multiple perspectives in mind, thus understanding that somebody else does not experience a situation in the same way as oneself. As repeatedly pointed out by mentalization theorists, these two stances of pre-mentalization are also common, temporarily or permanently, in adult patients with relational or personality difficulties (Fonagy et al., 2002; Bateman and Fonagy, 2016).

Based on the empirical data from patient interviews in the studies, I suggest in the model (Figure 1) that a patient with temporal or more long-lasting concrete or pre-mentalized thinking will have difficulties interpreting the therapist’s interventions, which is expressed in *patient experiences fusion between therapist’s personality and interventions*. Patients described their therapists by referring to their interventions and interpreted actions concretely, such as therapist silence as a sign that the therapist was “insecure” or “had problems of her own”. In line with this, the *patient cannot/dares not bring up criticism*, as doing so would equal to criticizing the therapist as a person. Also, one often overlooked difficulty for young patients is the subordinate position they might experience in relation to the older psychotherapist (Gibson and Cartwright, 2013), which could have posed a problem for patients in the present study, particularly in a pre-mentalizing mode.

Possibly, therapists in suboptimal therapies did not observe their patients’ sudden pre-mentalization, temporal or more permanent, or failed to address it in a fruitful way. Sudden shifts to pre-mentalization could be understood as the “state-like” part of the therapeutic alliance (Zilcha-Mano, 2017), i.e., a condition which changes (sometimes from moment to moment) over time. To observe and intervene would have helped the patient develop agency, a clearer self-understanding, reduce projection and lower anxiety over time (Allen, et al., 2008; Bateman and Fonagy, 2016). However, data from therapist interviews instead imply that therapists underestimated their patients’ problems and thus did not notice their lack of self-understanding and mentalization. Early in therapy, therapists established an image of the patient as competent, but later experienced that “only parts of the patients’ problems were brought into therapy”. To overestimate the patients’ functioning and underestimate their problems is generally correlated with lower outcome (Barber et al., 2013, p. 466). From this, it is reasonable to believe that better therapist ability to recognize sudden shifts in mentalization would help preventing suboptimal psychotherapy.

Patients with average outcome (study four) describe the development of *agency* during the course of therapy (“finding oneself and one’s way of life”), in contrast to those in suboptimal psychotherapy. Agency signifies the experience of a lasting identity or a self that has the ability to understand oneself and others and thus act in adaptive ways and is closely linked to the capacity to reflect upon situations, experiences and oneself in relation to others (Fonagy et al., 2002; Allen et al., 2008; Bateman and Fonagy, 2016). This lack of agency is what patients in suboptimal psychotherapy in the studies describe and ask for help with from their therapists. However, therapy did not help them develop this capacity. The interpretation of the process that lead to this stalemate is summarized to the right in the process model. The arrows upward and downward to the left depict the pathways to suboptimal psychotherapy or more successful psychotherapy, depending on the therapist’s actions.

The development toward suboptimal therapy with young adults is depicted by the arrow downward; *therapist does not discover/address* difficulties and pre-mentalization in the form of sudden changes of mentalization capacity or internal states, as described above. This leads to the circle of *no joint meta-communication*. The word meta-communication is used in this article to denote the explicit or implicit communication on the goals, tasks, and emotional bond in therapy (therapeutic alliance; Bordin, 1979), as well as communication on the moment-to-moment emotions and thoughts in therapy and the state-like therapeutic relationship (Zilcha-Mano, 2017). It is both the conscious, explicit verbal communication on this, and the implicit, non-verbal negotiation of alliance in therapy. Thus, it also includes implicit or affective communication and mirroring to a certain extent. The therapist’s role is to make room for this in the therapeutic collaboration by inviting the patient to share his/her impressions and views in therapy. It is the responsibility of the therapist to foster this and observe the patient’s capacity for meta-communication and adjust interventions. In itself, meta-communication is a mentalizing process, as the patient deepens her/his understanding of the self in relation to others (the therapist and other important persons) not just intellectually, but also with regard to affect. Thus, it is a capacity that the patient could develop during therapy, and a method to foster therapeutic alliance. As a capacity, it is similar to reflective function in mentalization theory (Bateman and Fonagy, 2016). Indeed, patients with personality disorders and low capacity for mentalization have expressed their wish for therapists who communicate clearly in the therapeutic setting (Morken et al., 2019), indicating that patients in the present study would have benefitted from such an approach as opposed to the restrained version of the psychoanalytic stance they described their therapists applied. When not noticing the sudden shifts in mentalization capacity, the *therapist underestimates the patient’s difficulties* which leads to *interventions not adjusted to patient’s level and needs*. The *patient does not understand the interventions*, neither the goals nor how to make use of them.

An example could be interpretations of how earlier experiences influence the patient’s current problems. If not rooted in present affects and emotional understanding in the here-and-now, such interpretations seem not to be useful for the patient according to this analysis. Patients in suboptimal therapies expressed that focus was too much on explanations from the past, which I understand as interpretations on a level the patient did not benefit from, although the therapists thought this to be useful. Focus on past experiences can be helpful if it is rooted in present emotions, which patients with better outcome in study four described. From the therapist’s perspective an overestimation of the patient’s capacity and functioning might contribute to the experience of “having only half of the patient in therapy,” as expressed by therapists in the primary analysis.

The *diverging agendas* in suboptimal therapy appear when the patient does not understand the therapist’s goals or interventions, and the therapist does not understand the patient’s difficulties and goals. When the patient and therapist do not have same agenda, therapy continues to be *pseudo-mentalizing: no corrective emotional experience* and *no increase in agency/self-understanding* develops. On the surface, therapy is centered on important issues, and the therapist might consider therapy helpful for the patient, whereas the patient does not experience positive change. The therapeutic alliance, in terms of shared goals, a common understanding of the tasks, and a good emotional bond (Bordin, 1979; Zilcha-Mano, 2017), is thus weak, which in turn makes it even more difficult for the patient to bring up criticism and share their experiences with the therapist. It is probable that the patient’s statement “spinning one’s wheels” in therapy without improvements (Werbart et al., 2015) referred to a therapy that was ostensibly reflecting, pseudo-mentalizing, but without affective content. As described by participants, the therapist and patient discussed parts of the patient’s life that might have been important, but the patient did not experience or feel that it made any difference, since it did indeed not make any affective difference. Patients in suboptimal therapy expressed better (intellectual) understanding but also that they did not feel any change. This could indicate a low integration of affect and thinking in the therapeutic process. Since mentalization is fostered in a secure relationship, this also implies the already suggested conclusion that the relationship to the therapist was not secure in attachment terms. An insecure relationship gave the patient less room for emotional exploration and corrective emotional experiences, since anxiety was easily awakened and difficult to regulate. As such exploration and activity in therapy was precisely what participants stressed as helpful, naturally therapies with low exploration would be less helpful.

In a rare study of patient experiences of non-improvement, Radcliffe et al. (2018) found that patients unfortunately had their negative self-image reinforced by the absent positive effects of therapy. The patients in their study started therapy with negative views of themselves and a fear of what psychotherapy might lead to (such as losing control), which might be understood as a fear of affects and a limited capacity to mentalize. Their therapists in the present studies did not become fully aware of this, indicating how difficult meta-communication is in practice.

The circle to the upper right depicts the positive process of joint meta-communication in a fruitful therapy, in particular when ruptures or stalemates appear. Even a very well-functioning therapy contains many instances of misunderstandings, difficult emotions the patient defends against, and disagreements, mainly referred to as ruptures (Barber et al., 2013). The repair of ruptures is seen as an important part of therapeutic change in relational psychotherapy (Safran and Muran, 2000) and mentalization-based interventions (Bateman and Fonagy, 2016), as it gives the patient a chance to take another person’s perspective (the therapist’s) and compare it to their own, thereby discovering own and others’ misinterpretations and projections. A large part of the patients in suboptimal therapy in the present studies were diagnosed with personality disorder, despite their young age, which indicates they suffered from relational dysfunction and would have needed therapeutic interventions adjusted to their needs. As their mentalization capacity seems to have fluctuated, they might have benefitted from therapy in which the therapist took a more active part in focusing on and solving alliance ruptures, for two reasons. Firstly, to increase their understanding of themselves and others (i.e., mentalization). Secondly, to create a secure emotional bond as far as possible. Judging by the data, none of this happened, although we do not have verbatim recordings of sessions to confirm it.

If the therapist discovers ruptures, and fluctuations in mentalization as they occur, the model proposes the development to a fruitful therapy is more likely. Participants in the studies described increased security when their unhelpful ways of thinking, as well as emotional difficulties, were addressed and confronted by the therapist. Meta-communication was then made possible, and patients gained increased self-knowledge and agency. As one patient expressed, the therapist “saw through” her and understood them on a deeper level, which was reassuring. Therapists in turn gained a clearer understanding of their patients’ needs and capacities, and could adjust interventions accordingly, which in turn made meta-communication easier. This is illustrated by the capture *patient experiences the relationship as secure*. In a secure enough relationship to the therapist, the *patient brings up criticism/diverging views* and also feels secure enough to explore these. When the patient shares her/his experiences, the *therapist understands and adjusts interventions* to the patient’s current level of functioning and mentalization. There is an *open negotiation of alliance* in terms of goals, tasks and the emotional bond, as well as implicit relational aspects of therapy. This leads to *increased agency/self-understanding*.

The model was developed to interpret the difference between a course of suboptimal therapy as a whole and a course of therapy with average outcome, based on the empirical data from the primary studies. However, a single course of therapy might also move back and forth between the circles on a micro-level, that is, in a single therapy session or in therapy at large. This is indicated in the model by the arrow between the two circles. An alliance rupture occurs for instance when the therapist does not understand the patient’s current limited mentalization capacity and makes abstract interpretations that the patient does not understand. Or another patient with relational difficulties might easily and quickly slip into a feeling of threat in therapy and regard everything the therapist says in this light. If the therapist notices this immediately, the slip can be immensely useful in the therapeutic work as a starting point for the exploration of situations in which the patient feels threatened.

### Alternative Interpretations

In the above interpretation, mentalization, and meta-communication play crucial roles, e.g., based on the patients’ concrete ways of expressing themselves, which is interpreted as pre-mentalizing modes. The secondary analysis should be seen as one of a number of possible interpretations and theoretical frameworks that could make sense of the fact that these therapies did not reach their goals.

What if the patients’ descriptions of the therapists and therapeutic processes should be taken at face value rather than analyzed. In other words, do the results describe an external reality of therapists who were inattentive during sessions, do they capture the patient’s inner mental representations of the therapist and relationship, or both? If so, observers would perceive the therapists as clearly too passive.

In psychotherapy research, the correlation between therapist warmth, engagement, agreeableness and flexibility in relation to good alliance and outcome has been stated (Barber et al., 2013). It is not surprising that participants in the studies described dissatisfaction when they experienced therapist passivity, ignorance, and powerlessness. One possible explanation for their descriptions would be that their therapies were indeed marked by high levels of criticism and negative comments from the therapists. Likewise, the patients’ call for an active therapist could be rooted in the therapist’s excessive silence, sleepiness, and an inflexible interest in childhood experiences, rather than relevant present emotions. Such an image would come close to the caricature of a psychoanalyst.

Although the primary studies were not designed to investigate therapist behavior, there are some points to be made. Even if an observer would conclude that therapists were negative and passive, the conclusion would not be sufficient from an interpersonal point of view. Passivity is not only a personal trait on one part, but also an interpretation of the therapist’s action made by the patient. Obviously the patients found the therapist’s lack of response hindering, and the therapist did either not perceive this or perceived it but did not change his or her approach. The question to be studied would then be how the interaction turned too silent and negative to be helpful for the patient and how the therapist could have found a way to handle this.

From an attachment perspective, the relationship between the patient and therapist can be viewed as an attachment relationship, and the therapist thus a potential attachment figure for the patient (Bowlby, 1988; Wallin, 2007; Slade, 2016). A secure attachment to the therapist would give the patient both a secure base and a safe haven, or, in other words, a balance between security and challenge in therapy. Individuals with an insecure attachment pattern more easily interpret others’ actions, remarks, and expressions as hostile or critical (Wallin, 2007). This raises interesting questions of how therapist and patient attachment patterns influence the perception of the communication, and thus the transference and counter-transference. The participants in the present studies of suboptimal therapy seemed vigilant for therapist presence and availability, much like an individual with insecure attachment checking the availability of their attachment figure (Wallin, 2007). Slade (2016) concludes that individuals with preoccupied attachments need interventions or therapies targeted to their interpersonal problems. Generally, patients with secure attachment orientation at the start of therapy seem to have better outcome and more easily form a good working alliance (Slade, 2016). One possible conclusion is that patients in suboptimal psychotherapies in the studies had more easily accessed insecure inner working models than others and would have needed this to be addressed, and would have needed encouragement from the therapist to bring up criticism and discontent in therapy.

However, a conclusion based on patient attachment style heavily relies on the patient’s part in the relationship. Therapists vary in their stance, skill and outcome (Baldwin and Imel, 2013). Thus, the question should be raised whether non-improvement and dissatisfaction were the result of a few therapists’ lack of good results. To follow-up results of the secondary analysis the project data on outcome of the therapists in the two studies of suboptimal therapies were revisited. In all, 19 therapists conducted the 24 suboptimal therapies. The majority of the therapists thus had only one patient who was dissatisfied or non-improved. The exceptions were one therapist with four non-improved patients, and two therapists with two patients each. However, these three therapists had a relatively large number of patients in the project at large compared to other therapists (six, five and seven, respectively), which means they also had patients who improved from their therapies. Thus, the suboptimal therapies were not solely the result of a few therapists’ low general outcome. It could still be possible that the combination of a specific participant and therapist was unfortunate, e.g., due to their attachment styles and the therapist’s inability to address this particular patient’s needs or deficit skills to meet this particular patient (Wallin, 2007).

Another possible interpretation of participants’ claim of therapist passivity is that the therapeutic interventions were not helpful, and thus experienced as therapist passivity. Gold and Stricker (2011) propose that psychodynamic psychotherapists should actively assess the patient’s level of functioning and should not avoid active interventions, if they are to reduce the risk of treatment failures. Traditional therapists might find this difficult (Gold and Stricker, 2011), which could have been the case in the present study. As an example of activity, psychodynamic affect focused therapy (e.g., McCullough et al., 2003) targets defenses, avoided affects and anxiety in relation to the affects in order to create changes in the way the patient perceives, experiences and expresses affects, leading to personality changes. Although we do not have process data to study interventions in the studies in detail, the conclusion that the interventions did not challenge defenses and help the patient experience authentic emotions and impulses is not too far-fetched. Participants’ descriptions of not feeling understood, or that therapy had the wrong focus, indicates that problematic emotions and defenses were not discovered or worked through in therapy. The participants’ call for therapist activity and lead could thus mean therapeutic action that would have helped them reach beyond defenses to a new understanding of their inner life. The participants expressed a wish for this in the interviews, but it did not come about in the therapies.

One possibility is that the match between therapist expectations or needs in therapy did not match the therapist competence or psychoanalytic psychotherapy, and that patients needed some other form of treatment such as CBT or pharmacotherapy.

Apart from mentalization theory, there could be other theoretical perspectives that would improve our understanding of the lack of success in the therapies that were analyzed and the processes involved.

To summarize, a look into therapist factors shows that therapists of the suboptimal therapies also conducted other therapies in the project with better outcome. Thus, the patients’ descriptions of therapist passivity and lack of response cannot only be explained by the general lack of skill of a few therapists in the project, although the therapists’ might have been passive with these particular patients. From an attachment perspective, the participants in suboptimal therapies might have had insecure inner working models, due to their attachment history, which contributed to their experience of therapist passivity, criticism, and unreliability. In line with this interpretation, therapists probably did not discover, address or find ways to work with attachment insecurity enough to create a secure therapeutic attachment relationship.

### Paradigmatic Example With Clinical Implications

As an illustration of the processes and theories involved in suboptimal psychotherapy presented in the tentative process model above, I present a fictive case. It is a combination of several cases from the suboptimal therapies in the empirical material, and is meant as a prototypical example without identifiable individual markers.

Amanda, 23, felt stressed out and “lost” when she sought therapy. She studied politics at a university but did not know whether to continue, took a term off and earned her living from time-limited employments. She moved between flats. The goals for therapy were mutually formulated: to be able to develop close relationships, feel secure in herself and know what she wanted. Therapy lasted 18 months and Amanda showed no improvement on the self-report measures of anxiety, relational functioning, and depression. Amanda expressed in the interview at termination that she experienced the therapist as uninterested in what Amanda tried to tell her, as the therapist was quiet and did not ask any questions of importance. Amanda tried her best to answer the few questions the therapist did ask, although she did not see the point. She thought the therapist knew best, as she was an experienced woman in her sixties. Amanda did not dare to bring up her dissatisfaction with therapy. Instead she blamed herself for not getting better. She could not reach her own feelings and did not feel comfortable with the therapist. However, the therapist was also kind, she expressed.

The therapist, a 62-year old female psychotherapist, teacher and supervisor with a background as social worker expressed in her interview how therapy with Amanda started well. They cooperated to find a common goal, and the relationship was good, but she sensed Amanda drawing back emotionally after a few months. The therapist asked questions she saw as important, but experienced Amanda as quiet or avoidant, which puzzled the therapist. Amanda often canceled late and the therapist was frustrated but found it hard to talk about, as Amanda avoided such questions. The therapist made an effort not to be obtrusive, since Amanda had described her mother as obtrusive, impulsive and temperamental, without respecting Amanda’s integrity. Amanda’s father, on the other hand, was described as unemotional and uncomfortable when Amanda tried to bring up anything important with him. The therapist saw emotional loneliness as part of Amanda’s problem and thought a focus on this would help Amanda.

As the example shows, the therapist and patient did not share the same image of the process in therapy, although both would agree that there were problems. In following the process model above, one could conclude that the patient was pressed by life decisions in young adulthood that had to be made but could not be made unless she knew her direction in life though a certain degree of agency. Amanda’s therapist thought she had difficult relations to her parents and an insecure attachment pattern, which the therapist wanted to work on and tried to explore with Amanda. Amanda, however, “did not see the point” in these interventions but still answered the therapist’s questions, possibly partly because of the age gap between the two which made Amanda think the therapist was a wise person. We cannot say with certainty that Amanda often reasoned concretely or in pre-mentalized modes, but it is obvious that she did not dare to express criticism, which then made the therapist unaware of her perception of the therapist. This led to a vicious circle of mutual misunderstanding. There seems to have been no meta-communication. Obviously, the therapist sensed some of the interpersonal difficulties Amanda had in relation to the parents, but did not discover or address her submissiveness in therapy. Neither did the therapist explore the areas Amanda considered most important, which were her direction in life in terms of education and employment. In a focus on those areas, Amanda could have had a good chance of developing agency.

From the perspective of the present model, it is possible that the therapist would have needed to be observant on the patient’s present emotional state and capacity to mentalize in order to move from the suboptimal circle to the positive circle within a session with Amanda. One way is to bring up difficulties in the relationship in a responsive way, to meta-communicate and build a secure relationship with Amanda. Interventions for this are described within a number of therapeutic theoretical orientations. One is to continuously assess the patient’s affects, not only at therapy intake, but during therapy, as it shifts from moment to moment, for instance through interventions developed in affect-focused psychotherapy (McCullough et al., 2003). By explicitly labeling the patient’s emotional states or reactions (i.e., “I see you turn away your gaze when I ask you about this, I wonder what is going on inside you”), the chances for meta-communication would have increased. It would also have helped the patient become aware of her own reactions and possibly understand herself better. The point of such interventions is to draw the patient’s attention to his or her emotions and explore them together with the therapist to develop the mentalized affectivity mentioned earlier (Bateman and Fonagy, 2016).

The therapist could have initiated meta-communication on the therapeutic bond in general and in the present moment (Bordin, 1979; Zilcha-Mano, 2017), for instance by asking what the patient thought about her experiences of therapy. In the case of Amanda, the therapist could have meta-communicated in by sharing her thought, saying “I sometimes wonder if you feel it helps to talk to me. I think it is sometimes helpful, but I also doubt it from time to time, especially when we talk about your relationship with your friend.” Or, since patients still might hesitate to express criticism, feedback can be formalized as questionnaires on therapeutic alliance. The trait-like alliance (Zilcha-Mano, 2017) is similar to internal working models in attachment theory (Bowlby, 1988) and could be assessed formally or informally. Amanda’s expectations might have been that closeness is often conditioned by the other person, and could be intrusive. State-like alliance is contextual and changes with the situation and therapeutic action, i.e., the therapist’s understanding, or lack thereof. Amanda had repeated experiences of ruptures in the relationship and seems to have reacted by withdrawing. A responsive and careful focus on this pattern would most likely have been helpful meta-communication in her case and might have turned the non-improvement into fruitful therapy.

In line with this, openness to the patient’s sudden changes in mentalization level might have been helpful, for instance when Amanda perceived the therapist as uninterested. Such interventions help the patient become aware of their own emotional state and thus also of possible projections onto the therapist of hostility or disinterest, which makes criticism easier to bring up in therapy. Since affect awareness and mentalization are under development for young adults, interventions with such a focus bear the potential not only for giving room for criticism from the patient, but for a healthy development of mentalization and self-reflection in itself.

A way for the therapist to develop therapeutic skills fostering meta-communication is to enhance his or her own ability to discover and address concrete pre-mentalized modes of thinking, e.g., by professional development and supervision on difficult cases. Therapists have their personal challenges or difficulties with certain patient cases or emotions. The awareness of this and the use of supervision when needed, possibly in a form targeted towards these particular difficulties, such as deliberate practice (Rousmanière, 2016), is one way to help oneself as a therapist.

To conclude, there are interventions that foster joint meta-communication and a secure therapeutic relationship with young adults. Such interventions generally include both assessment and meta-communication at the start of therapy, as well as during therapy. More detailed descriptions on developing skills to meet the challenges of young adult patients, and patients where developing a therapeutic alliance is difficult, need to be formulated within each therapy tradition or orientation.

### Bridging the Gap Between Practice and Research

The results and discussion raised a number of clinically relevant questions. I summarize some of them here in the form of questions and answers. These clinical conclusions are meant to be easily accessible, based on results from the studies and previous research. By necessity they are held simplified, short and general. The present studies are based on psychoanalytic psychotherapies, and most of the research cited is either psychodynamic in orientation or generic. Thus, the conclusions might not be valid in other contexts.

#### What Do These Studies Tell us About Suboptimal Psychotherapies From the Patients’ Perspective?

Even therapists with long experience and good results with other patients might have therapies in which patients do not improve or are dissatisfied. It might be difficult for therapists to discover non-improvement or dissatisfaction. In the studies, therapists of non-improved patients overestimated their patients’ capacity and underestimated their difficulties.

#### How Come Patients Do Not Bring up Criticism With Their Therapist?

It is well-known that patients refrain from criticizing their therapists, or do so very reluctantly. Many psychotherapy patients, and probably in particular those in suboptimal therapies, see the therapist’s interventions and personality as a whole. Criticizing lack of progress in therapy or the therapist’s interventions thus amounts to criticizing the therapist personally.

#### How Could Therapists Encourage Patients to Bring up Criticism?

By making room for it in a way that suits the therapist, patient and therapy method. Meta-communication on the relationship between the therapist and patient, as well as the interventions in therapy, is one way. That is, the therapist routinely asks for the patient’s views on how the two are getting along and reactions to interventions. Therapists could also routinely check therapy progress and address any deviations in therapy by using standardized measures for symptom relief and therapeutic alliance. They could also pay attention to the continuous assessment of the patient’s emotional and relational functioning throughout the therapy, in order to discover ruptures. If the therapist senses that the relationship does not feel right, they could bring it up.

#### What Do Patients Usually Bring up When Researchers Ask Them What They Did Not Like in Their Therapies, and What Do We Learn From it?

There might be differences in criticism across therapy orientations. The studies in this article concerned psychoanalytic psychotherapy. Often, patients suggest that they and their therapist had different perspectives on the goals and tasks in therapy. That is, they did not agree on how to best use the time in therapy. This might not have been outspoken. In the therapies of these studies, goals, and methods were discussed, but patients still experienced focus was partly on the wrong things. Furthermore, they did not seem to be secure enough in the relationship to be able to criticize the therapist. Moreover, patients brought up that their therapists were not active enough, which could be interpreted as not enough initiative to target the most important issues. Therapists might need to observe more closely when the patient needs further pedagogical explanations of therapeutic method in the beginning of therapy.

#### What Is There to Do When the Relationship Between Therapist and Patient Does Not Feel Right or a Patient Does Not Seem to Get Better?

There will be ruptures often in therapy. There are interventions that focus particularly on ruptures in the therapeutic alliance, and difficulties in the relationship, in therapies of different theoretical orientation. The aim of such interventions is to create a secure atmosphere where criticism and difficulties can be brought up. It can then be used in therapy as a way of understanding and working with the patient’s interpersonal difficulties. Since patients seem not to differentiate between technique and therapist personality when things go wrong, working with the relationship and the moment-to-moment interpersonal situations in therapy might be helpful. Sometimes a change of therapist might be considered, if the issues are hard to solve. Also, to bring up the question of improvement, to see whether patient and therapist agree that there is no expected improvement is of importance. In these studies, patients seemed to have an idea of what would help them, but in suboptimal therapies it was most likely not discussed with the therapist. If the therapist is able to help the patient make the idea of what would be helpful explicitly, and compare these to their own views of therapy, it will be easier to know when the therapist should recommend a change of therapy.

#### What Is Special About Young Adult Patients?

The results suggest one central issue is agency. This includes developing a sense of identity which is stable across many situations and an awareness of one’s own will and feelings. Also, the therapist needs to practice responsiveness for the therapeutic alliance to be able to meet the patient. The therapist could remind herself that young adults have limited experience of themselves in different situations, since the capacity for mentalizing and reflection is still developing.

#### Final Conclusions on Research and Clinical Practice

As an example of how research results and clinical practice might influence each other, I present the intertwined clinical and theoretical conclusions that can be drawn from this study as I have come to use them. My professional development as a psychotherapist during the course of the project influenced and was influenced by the research results. While conducting psychotherapy with young adults, I experienced how difficult it is for a therapist to discover patient dissatisfaction or patient experiences of not being understood and how often I did not succeed. The research results have encouraged me to focus on the three parts of the therapeutic alliance (Bordin, 1979) when first meeting the patient. Few patients have a clear image of what means of therapy they wish for, but most have thought about the goals and all of them can express how the therapeutic relationship develops when asked. I try to explain as clearly as possible what goals I consider realistic and how I would understand these therapeutically. I invite patients to bring up doubts and negative aspects of the therapeutic relationship in order to work with their view of it, which is not the same as trying to change according to their wish, but rather a means to bring up transference and misunderstandings at an early stage.

I focus on alliance ruptures whenever I discover them. Sometimes they are resolved, sometimes not. I do not presuppose that my and my patient’s view of the ruptures and therapeutic process in general coincide, but I ask patients regularly how they experience we are getting on.

Patients with severe personality difficulties typically need more support and treatment than a therapist in private practice can offer, which calls for good assessments at the beginning of therapy.

Lastly, in order to prevent patients from entering or continuing a therapy in which we would only be spinning our wheels, I have learnt to more often bring up the question of whether a therapist of another orientation would be better suited to help the patient in some cases.

### Strengths, Limitations, and Future Directions

The naturalistic setting meant that therapies were studied as they were conducted at the Institute of Psychotherapy in Stockholm, which in general led to good external validity and translation to a clinical setting. The longitudinal design added further naturalistic value of the data. There were measure points before therapy, at termination of therapy, and at two follow-ups, 18 and 36 months after termination, which is a substantial time in psychotherapy research. Additionally, to adopt the double perspectives of the patient and therapist was a strength, as was the mixed method design (Elliott et al., 1999; Creswell, 2011; Teddlie and Tashakkori, 2011) that highlighted non-improvement and dissatisfaction from different angles. A mixed method approach is useful from pragmatic standpoints and can be applied by combining quantitative and qualitative data and analyses in different stages of the process (McLeod, 2013). The naturalistic setting had the disadvantage of non-manualized treatments. However, the aim was to study a natural setting, and non-manualized treatment or manualized treatment without adherence measures constitute the reality in many clinics. A major limitation was that no recordings of sessions could be done, which limits the conclusions of the process of the therapies as it could not be observed, but interpreted retrospectively. The design and data do not make room for any causal conclusions. The primary and the secondary analyses should rather be seen as an interpretation of the qualitative data in the light of mentalization with the aim to suggest hypotheses for further research and improvements in therapeutic practice.

The participants of the studies were generally from urban, well-educated middle class areas, which limits the transferability (Lincoln and Guba, 1985; Malterud, 2001b). Emerging adults in small towns might express different wishes for psychotherapy and also face different challenges in life in general. One limitation was the overlap of four patients, who took part in more than one of the primary studies (three were dissatisfied and non-improved, two non-improved with a depression diagnosis, one of was also dissatisfied). However, grounded theory aims to analyze themes on a group level, and the participant who occurred in all three studies thus had a limited influence on each model as a whole.

All studies aimed for credibility in qualitative terms (Elliott et al., 1999; Malterud, 2001b; Morrow, 2005). In all primary analyses the emerging categories and core concepts were discussed between the researchers involved in each study, which is a form of triangulation (Charmaz, 2014). The researchers in each study explored rivaling interpretations of data such as whether a subgroup of therapists had particularly low outcome, as well as alternative tentative models and categories throughout the analyses as a part of the constant comparative analysis (Charmaz, 2014). In the secondary analysis, the author discussed emerging categories and process models with one of the authors of the other articles (Andrzej Werbart) as well as research teams at the department. The process model was re-formulated several times. Concerning transferability, or the degree to which the results can be useful for other contexts than the one studied, the naturalistic design meant circumstances were similar to many psychoanalytic clinical contexts in terms of patient inclusion, formulation of goals in therapy and presenting problems among patients. Thus, results can be expected to be relevant for some contexts outside the research setting.

The interpretation in the shape of the tentative process model in a theoretical interpretation, and data could possibly also be interpreted by using other theoretical frameworks, in line with the hermeneutic strand of psychotherapy research (Rennie, 2006). However, the usefulness of mentalization theory in the present article shows its strength in describing the vicissitudes of the psychotherapeutic process that does not lead to the desired change.

The need for studies of patients’ understanding of suboptimal psychotherapy needs to continue in order to prevent it. The study of ruptures on a micro-level is a growing field (Zilcha-Mano, 2017, 2019), but the research is often limited to adults or not does differentiate between young patients and adults. Young patients have a mentalization capacity still under development, and research focusing on how to let this knowledge inform psychotherapy interventions is needed.

## Data Availability Statement

The datasets generated for this study will not be made publicly available. Patients could be identified if the interviews (raw data in this article) are publicly available. Also, patients’ consent did not include such public availability. The parts of data which can be anonymized are available from the corresponding author on request, after discussion.

## Ethics Statement

The studies involving human participants were reviewed and approved by Regional Research Ethics Committee at Karolinska Institutet. The patients/participants provided their written informed consent to participate in this study.

## Author Contributions

The author confirms being the sole contributor of this work and has approved it for publication.

## Conflict of Interest

The author declares that the research was conducted in the absence of any commercial or financial relationships that could be construed as a potential conflict of interest.
